# Utility of microbial cell-free DNA sequencing in the diagnosis of mycobacterial infections in a quaternary care center

**DOI:** 10.1017/ash.2025.10057

**Published:** 2025-07-17

**Authors:** Fernando H. Centeno, Todd Lasco, Ahmed M. Hamdi, Mayar Al Mohajer

**Affiliations:** 1 Department of Medicine, Baylor College of Medicine, Houston, TX, USA; 2 Department of Pathology and Immunology, Baylor College of Medicine, Houston, TX, USA; 3 Baylor St. Luke’s Medical Center, Houston, TX, USA

## Abstract

We examine the performance of microbial cell-free DNA (mcfDNA) next-generation sequencing (NGS) testing on patients admitted to a quaternary care hospital in Houston, Texas. The test was 75.0% sensitive and 97.8% specific for all mycobacterial infections. mcfDNA NGS results led to adjustments in antimicrobial therapy for seven of nine patients with positive results.

## Introduction

Mycobacterial diseases remain a substantial cause of morbidity and mortality in the United States, where the prevalence of tuberculosis (TB) is 4.4–4.8%.^1^ The prevalence of non-tuberculous mycobacteria remains more infrequent but persists at 11.70 per 100,000.^2^ Establishing the diagnosis in these cases promptly is challenging and often requires the evaluation of multiple, time-consuming, quality-dependent tests that may not definitively rule out the infection.^3^

Next-generation sequencing (NGS) of microbial cell-free DNA (mcfDNA) has become increasingly common as an open-ended method for testing against a broad range of pathogens through non-invasive plasma sampling.^4^ Previous studies of mcfDNA assays have characterized the sensitivity and specificity of the test in TB cases.^5^ Less clear is the role mcfDNA NGS plays compared to other diagnostic modalities, its performance, and its impact on management. In this retrospective case series, we characterize the mcfDNA role in mycobacterial diagnosis and management in one quaternary medical center.

## Methods

The performance of mcfDNA NGS was compared to mycobacterial cultures among patients who had both tests performed between 2017 and 2024. The electronic medical records for patients with positive mcfDNA NGS for mycobacteria were examined for clinical symptoms, radiology, diagnostic tests, and clinical management.

mcfDNA NGS tests were sent to Karius, Inc. (Redwood City, CA), the sputum acid-fast bacilli (AFB) PCR was sent to Quest Diagnostics ® (Secaucus, NJ), while the mycobacterial cultures were performed in-house.

The primary outcome was the sensitivity of the mcfDNA NGS tests compared with mycobacterial cultures. The secondary outcomes included the turnaround time (TAT) and whether mcfDNA NGS led to a change in antimicrobial management. The clinical TAT was defined as the time from physician order and collection to reporting results (days). In contrast, the laboratory TAT was defined as the time from specimen receipt by the laboratory to reporting results. This study was approved by the Baylor College of Medicine Institutional Review Board.

## Results

149 patients had both mcfDNA NGS and mycobacterial cultures performed, of which 135 were positive for neither, and 15 were positive for at least one. Five patients with Mycobacterium tuberculosis (MTB) on mcfDNA NGS had confirmatory cultures. Four had non-tuberculous mycobacteria (NTM) on both methods, and three had positive cultures but negative NGS. Three others had positive NGS for NTM but negative AFB cultures, with two deemed false positives and one with a prior history of Mycobacterium avium complex (MAC). Sensitivity and specificity for mcfDNA NGS were 75.0% and 97.8%; for MTB, both were 100%, while NTM sensitivity was 57.1% and specificity 97.8%.

Nine patients with positive mcfDNA NGS and microbiologic data were reviewed: five for MTB and four for NTM. The median age was 69 years, with six males and three females. Seven had risk factors for mycobacterial infection, including two from endemic regions, one on adalimumab, a lung transplant recipient, and three with AIDS (CD4 < 50). Chronic fever and weight loss were common, with lymphadenopathy seen in three MTB and two MAC cases.

On average, each patient received 27 diagnostic studies for infectious diseases (range 14–78) before diagnosis (Table 2). All patients except one had pending tests for mycobacterial disease at the time mcfDNA NGS was sent, one already had a positive culture for *Mycobacterium abscessus*, and one had a previously diagnosed MAC infection.

**Table 2.** Imaging and laboratory studies for study patients

Laboratory studies	1	2	3	4	5	6	7	8	9
AFB-Stained Smears^a^	CSF: No AFB seen in 3 samples. Sputum: No AFB seen in 1 sample	Esophageal biopsy staining for acid-fast bacilli: Positive Sputum: AFB seen in 1 of 4 samples	Sputum: No AFB seen in 8 samples	Lymph node biopsy: AFB seen^b^ Sputum: No AFB seen in 2 samples Lung biopsy: No AFB seen Blood: No AFB seen Retroperitoneal biopsy: No AFB seen	Blood: No AFB seen Bone marrow: No AFB seen	Sputum: AFB seen in 1 of 4 samples^b^	Bronchial wash: AFB in 1 of 24 samples Blood: No AFB seen	Lung biopsy: No AFB seen	Stool: No AFB seen
Mycobacterial cultures and time to from collection to results^a,c^	Bronchioalveolar lavage: Positive for MTB Cerebrospinal fluid: Positive for MTB	Sputum: Positive for MTB Blood: Negative	Bronchial sputum: Positive for MTB Blood: Negative Bone marrow: Negative	Bronchioalveolar lavage: Positive for MTB Blood: Positive for MTB	Blood: Positive for MAC ^b^ Bone marrow: Positive for MAC	Sputum: Positive for MTB	Bronchioalveolar lavage: *Mycobacterium abscessus* ^b^ Blood: *Mycobacterium abscessus*	Lung biopsy: *Mycobacterium intracellulare*	Stool AFB culture: MAC ^b^
PPD/IGRA	Negative	Borderline	Not done	Invalid (failed positive control on IGRA)	Not done	Not done	Not done	Not done	Not done
Other mycobacterial tests^a,c^	Sputum MTB PCR: Positive CSF MTB PCR: Positive	Sputum broadband PCR: positive for MTB	Not done	Not done	Not done	Not done	Bronchioalveolar lavage MTB PCR: negative	Not done	Not done
NGS MPM for Mycobacteria (RR <10)^d^	277 (MTB)	4787 (MTB)	Positive (MTB)^e^	Positive (MTB)^e^	Positive (MAC)^e^	388 (MTB)	Positive (*M. abscessus*)^e^	45 (MAC)	>316,000 (MAC)^f^
NGS collected	Day 8	Day 12	Day 13	Day 4	Day 9	Day 5	Day 50	Day 12	Day 2
NGS received	Day 9	Day 13	Day 14	Day 8	Day 13	Day 6	Day 52	Day 14	Day 4
NGS reported	Day 10^b^	Day 14^b^	Day 15^b^	Day 10	Day 14	Day 8	Day 54	Day 15^b^	Day 5
Anti-mycobacterial treatment start date	Day 9	Day 14	Day 16	Day 9	Day 10	Day 4	Day 50	Not started	Started prior to admission
Antimicrobials stopped after diagnosis	Vancomycin, meropenem, amphotericin B	None	Vancomycin, meropenem, micafungin	Azithromycin	None	Azithromycin	None	None	Meropenem
Other NGS Results	None	EBV: 224	None	EBV^e^, Torque teno virus 3^e^	CMV^e^	None	CMV^e^	None	None
Days from admission to diagnosis	10	14	15	8	12	4	32	15	Diagnosed prior to admission
Number of infectious diagnostic studies	41	18	25	25	19	11	78	15	14

Abbreviations: CSF, Cerebrospinal fluid; AFB, Acid-fast bacilli; MTB, *Mycobacterium tuberculosis*; MAC, *Mycobacterium avium* complex; PPD, Purified Protein Derivative; IGRA, Interferon Gamma Release Assay; NGS, Next-generation sequencing; MPM, Molecules per microliter; RR, Reference range; EBV, Epstein-Barr virus; CMV, Cytomegalovirus.

a Dates are reported from day of hospital admission. Dates in which tests were received are reported for send-out studies.

b First positive mycobacterial diagnostic result received for a patient.

c Patients received multiple cultures and repeat PCR tests after the ones included in the table, as reflected in the counts of laboratory studies. For conciseness, only the first set are shown in this table.

d In a 684 healthy subject cohort used in the Karius test validation process, the 97^th^ percentile of both *Mycobacterium tuberculosis* and *Mycobacterium avium* complex mcfDNA concentration was 10 MPM (Blauwkamp et al., 2019)

e Some patients seen in 2017 received an earlier version of mcfDNA NGS studies that did not quantify the MPM of mcfDNA. Instead, the samples were tested with a negative buffer control.

f Concentration above quantifiable range for the assay.

The median clinical TAT for mcfDNA NGS was 3.3 days (range 2–6), while the median laboratory TAT was one day (range 1–2). Mycobacterial cultures had a median clinical and laboratory TAT of 27 days (range 9–46). Three acid-fast stains were positive: a lymph node biopsy and two sputum specimens with a median clinical and laboratory TAT of one day. Two patients had a positive sputum MTB PCR with a clinical TAT of nine days for both and a median laboratory TAT of 4.5 days (range 2–7).

The median time from admission to diagnosis was 11 days (range 4–15). In four patients, NGS results were the first to be positive. In three, alternative testing resulted in positive for MAC and TB before NGS results were received. The diagnosis was already established in two when mcfDNA NGS testing was sent.

Anti-mycobacterial therapy was started empirically in three patients: two with tuberculosis and one with NTM. One was already receiving therapy for a prior MAC diagnosis. In two, anti-mycobacterial therapy was started once positive NGS had resulted. In two, treatment was started after positive mycobacterial cultures. One was discharged and lost to follow-up before the start of treatment. Additionally, five patients discontinued empiric antibiotics (vancomycin, meropenem, amphotericin B, micafungin, and azithromycin) once positive NGS results were received. Four of nine patients required intensive care unit admissions and intubation. One patient expired, one was discharged to hospice care, and another to a skilled nursing facility, while the rest were discharged home.

## Discussion

Our results showed that when clinical suspicion is high, mcfDNA NGS testing can be an additional and valuable tool to achieve an accurate and timely diagnosis. Despite being a send-out test collected after other mycobacterial studies, mcfDNA NGS testing established the diagnosis before other laboratory studies in four out of nine patients. It also led to changing clinical management in patients due to earlier initiation of antimycobacterial treatment and discontinuation of other antimicrobials.

There were significant issues encountered by clinicians when using traditional tests, including the time required for laboratory diagnosis using conventional mycobacterial culture and send-out TB PCRs and the limited sensitivity of acid-fast smears. NGS of mcfDNA allowed clinicians to discontinue unnecessary antibiotics in some patients and start anti-mycobacterial therapy in others. The observed performance of the test was greater for MTB than seen in a prior study, ^5^ but it was markedly less for NTM than MTB. Notably, the delay in ordering the mcfDNA NGS compared to traditional tests could have negatively impacted its performance.

The test results must be carefully correlated with clinical presentation, as it can detect low concentrations of organisms like MAC that may colonize airways without causing disease. Additionally, mcfDNA NGS remains costly at $5,494 compared to $231 for MTB PCR and $4,325 for daily ICU charges. Until costs decrease, this test should be reserved for cases where diagnostic delays may lead to clinical deterioration and increased spending.

The greatest benefit of mcfDNA NGS was observed when traditional tests were done alongside NGS to maximize microbial identification and expedite results or when the disease presentation was nonspecific and broad antimicrobial therapies were used. Patients where mcfDNA NGS did not change outcomes were often immunocompromised, with the disseminated disease diagnosed by sampling sputum, blood, or stool. A prior study showed no clinical benefit in over nine of ten tests. Clinicians should carefully assess available diagnostics and their potential impact on outcomes.^7^

While this series does not definitively answer how diagnostic approaches for such complex presentations should be adjusted, it does illustrate the necessity for further investigation to best utilize this open-ended diagnostic tool in such challenging encounters. Our study is limited by the small number of patients included. Also, the TAT of different laboratory tests varies by the institution and microbiology laboratory capacity, affecting the generalizability of our findings.

**Table 1.** Clinical characteristics of study patients

Presenting features and imaging	1	2	3	4	5	6	7	8	9
Age, sex	60, female	69, male	73, male	56, male	23, male	71, female	73, male	75, female	32, male
Initial diagnosis	Pneumonia, later readmitted for seizure	Gastrointestinal infection or malignancy	Acute on chronic congestive heart failure	Sepsis	Fungal, mycobacterial, or viral infection vs malignancy	COPD exacerbation and possible pulmonary tuberculosis	Suspected pneumonia	Lung malignancy	Disseminated *Mycobacterium avium* complex
Final diagnosis	Pulmonary tuberculosis and tuberculous meningitis	Pulmonary and gastrointestinal tuberculosis	Pulmonary and disseminated extrapulmonary tuberculosis with bone marrow infiltration	Pulmonary and miliary tuberculosis	Disseminated *Mycobacterium avium* complex with bone marrow infiltration	Pulmonary tuberculosis	Disseminated *Mycobacterium abscessus* infection	Cavitary lung mass due to *Mycobacterium avium* complex	Disseminated *Mycobacterium avium* complex
Contact history/TB risk factors	Adalimumab, smoking tobacco	Immigrant from tuberculosis-endemic area, alcoholism	Immigrant from tuberculosis-endemic area	AIDS (CD4 count: 25 cells/mm^3^), cocaine abuse	AIDS (CD4 count: 9 cells/mm^3^), intravenous drug abuse	None	Recent lung transplant on mycophenolate, tacrolimus, and methylprednisolone	None	AIDS (CD4 count: 6 cells/mm^3^)
Past medical history	Psoriatic arthritis, pulmonary embolism	Radiculopathy	Congestive heart failure	HIV	HIV	Chronic obstructive pulmonary disease, remote ovarian cancer requiring hysterectomy	Interstitial pulmonary fibroris requiring bilateral lung transplant, subsequent pulmonary embolism, encephalopathy	Metastatic renal cell carcinoma, pulmonary embolism	HIV
Fevers	102°F	102°F	101°F	102°F	101°F	100.7°F	101.1°F	None	None
Cough	No	No	Yes	Yes	No	Yes	No	Yes	Yes
Weight loss	Yes	Yes	Yes	Yes	Yes	Yes	No	No	Yes
Lymphadenopathy	Yes	Yes	No	Yes	Yes	No	No	No	Yes
Presenting Chest X-Ray Report	Interstitial opacities, no focal consolidation	Not done	No focal consolidation, pneumothorax, large pleural effusion, or evidence of overt pulmonary edema	No acute abnormality	No acute abnormality	Left perihilar and middle and upper lung parenchymal pleural opacity worrisome for bronchogenic or metastatic malignancy	Hazy bilateral airspace opacities	5.2 cm cavitary mass in right mid-lung and subpleural nodules	No acute abnormality
Presenting Computerized Tomography Report	Chest: Diffuse reticulonodular opacities bilaterally with upper lobe predominance; mildly enlarged mediastinal and hilar lymph nodes	Chest: Lower esophageal mass with mediastinal, right hilar and left supraclavicular metastatic disease; Many pulmonary micronodules predominantly in posterior right upper lobe	Chest: Ground glass opacities in both lungs; trace bilateral pleural effusions. Nonspecific fluid collection inferior to left ventricular assist device	Chest: Multiple pulmonary nodules, largest measuring 4 mm Abdomen: Prominent upper abdominal and retroperitoneal lymph nodes	Chest/abdomen/pelvis: Prominent lymph nodes in mediastinum and abdomen without pathologic enlargement; marked splenomegaly, small right liver lobe lesion	Chest: Multiple calcified granulomas and left upper lobe granulomas; extensive bilateral opacities including reticulonodular opacities	Chest: Bilateral ground glass and patchy airspace opacities	Chest: Increase in size of cavitary lesion, now 7.5 x 4.0 cm and right pleural effusion	Abdomen: Extensive abdominal lymphadenopathy and splenomegaly with innumerable hypodense lesions concerning for neoplasm. Small bowel wall thickening. Ascites
Intensive Care Unit Admission	Yes	No	Yes	No	No	No	Yes	No	Yes

*Abbreviations*: TB, Tuberculosis; AIDS, Acquired immunodeficiency syndrome; CD4, Cluster of differentiation 4; HIV, Human immunodeficiency virus.
